# Alpha thalassemia deletions found in suspected cases of beta thalassemia major in Pakistani population

**DOI:** 10.12669/pjms.332.11834

**Published:** 2017

**Authors:** Saba Shahid, Muhammad Nadeem, Danish Zahid, Jawad Hassan, Saqib Ansari, Tahir Shamsi

**Affiliations:** 1 Saba Shahid, PhD. Department of Genomics, National Institute of Blood Disease & Bone Marrow Transplantation, Karachi, Pakistan; 2 Muhammad Nadeem, FCPS (Haem). Department of Hematology, National Institute of Blood Disease & Bone Marrow Transplantation, Karachi, Pakistan; 3 Danish Zahid, M.Sc Genetics, Department of Genomics, National Institute of Blood Disease & Bone Marrow Transplantation, Karachi, Pakistan; 4 Jawad Hassan, FCPS (Haem). Department of Hematology, National Institute of Blood Disease & Bone Marrow Transplantation, Karachi, Pakistan; 5 Saqib Ansari, PhD. Department of Hematology, National Institute of Blood Disease & Bone Marrow Transplantation, Karachi, Pakistan; 6 Tahir Shamsi, FRC Path. Department of Hematology, National Institute of Blood Disease & Bone Marrow Transplantation, Karachi, Pakistan

**Keywords:** Alpha thalassemia, Beta thalassemia major, Co-inheritance, Genetic analysis, α^3.7^, HBH disease

## Abstract

**Background & Objective:**

Alpha (α) thalassemia is a hereditary disorder and is caused by deletions or mutations in globin genes. It is present in two clinically significant forms: hemoglobin Bart hydrops fetalis (Hb Bart) syndrome and hemoglobin H (HbH) disease. It is highly prevalent in South-East Asia or Mediterranean countries. The most common deletion reported in alpha thalassemia in Pakistani population was –α^3.7^ with a frequency of 8.3%, and the rare forms were –α^4.2^ (0.2%) and ααα^anti3.7^ (0.9%). In our study, diagnosis of severe anemia cases without any α and β mutations or deletions were made by using extended alpha thalassemia deletions panel. The main objective of this study was to determine the prevalence and to study the spectra of alpha thalassemia gene deletions in beta thalassemia patients with the use of an extended panel including −−^SEA^, −−^FIL^, −−^MED^, −−^20.5^, −−^THAI^ in addition to –α^3.7^, –α^4.2^ & -ααα^anti3.7^.

**Methods:**

The samples were collected in ethylenediaminetetraacetic acid (EDTA) vacutainers. A total of 156 samples were analyzed for alpha thalassemia mutations. This cohort included 121 samples of beta thalassemia major, nine samples of beta thalassemia minor and 26 without any evidence of beta thalassemia mutations. DNA was extracted with Qiagen extraction kit. The primers for determination of different subsets of alpha thalassemia deletions were included. PCR amplification was performed and result interpreted on agarose gel.

**Results:**

Co-inheritance of alpha thalassemia (–α^3.7,^ –α^4.2^) with homozygous beta thalassemia was detected in 30% cases of studied cohort (37 out of 121). The most common found was –α^3.7^ deletion (35/37) as single/double deletions or in combination with -ααα^anti3.7^. In undiagnosed cases screened for beta thalassemia major, we found Mediterranean (–α^MED^) deletion at specifically 875 bp on agarose gel. This is distinctive finding in case of detecting –α^MED^ instead of any other deletion from Pakistan.

**Conclusion:**

Alpha thalassemia deletions (–α^3.7,^ –α^4.2^) are the common co-inherited deletions found in beta thalassemia major patients. On the basis of results, we propose an extended alpha thalassemia genetic mutation panel should be used for screening of children presenting with anemia with suspicion of haemoglobinopathy.

## INTRODUCTION

Alpha (*α*) thalassemia is a monogenic disease that characterized by decrease or absent synthesis of the *α*-globin chains due to deletion or mutation in *α*-globin gene. It is prevalent in South-East Asian, Middle Eastern and Mediterranean population but intermediate and severe forms are rare in North America and Northern Europe.^1^,^2^ Multiple types of deletions or mutations have been found to influence the clinically observed heterogeneous phenotypes of α-thalassemia cases.^3^

In an individual two *α* genes are located on each chromosome 16 (αα/αα). The loss of one (-α/αα), two (-α/-α or --/αα) and three (--/-α) gene are commonly cause *α*-thalassemia.^4^ The most common single gene deletions in *α*-thalassemia are the –α^3.7^ and the –α^4.2^, while the double gene deletions in cis, such as the −−^SEA^, −−^FIL^, −−^THAI^ alleles are most common in the South-East Asia and the —^MED^ and ––*α*^(20.5)^ double gene deletions occur most frequently in the Mediterranean area.^5^ In Pakistani population the most common deletion reported is –α^3.7^ with a frequency of 8.3%, and the rare forms are –α^4.2^ (0.2%) & -ααα^anti3.7^ (0.9%).^6^ Deletion of both *α*-globin genes on one chromosome are less frequently caused by a point mutation.^7^ Patients with non-deletional *α*-thalassemia have a more severe phenotype in contrast with the deletional type.^8^-^10^

Mutations in *α*-globin gene also play an important role in the pathophysiology and clinical severity of β-thalassemia (β-thal). When β-thal mutations in heterozygous/homozygous state coexists with α-gene alteration, the clinical and hematological phenotype of thalassemia could change to mild anemia in case of α deletion (-α/αα) or causes severe anemia in case of α triplication (αα/ααα).^11^ The diagnosis and management of *α* thalassemia may be complex by the heterogeneity of diseases, which is due to the interaction of co-inherited *α*-thalassemia with the variable severity of β-thalassemia mutations.

α-thalassemia carriers carrying single-copy deletion of the globin gene are clinically presented with a very mild hypo chromic microcytic anemia. Hemoglobin Bart hydrops (Hb Bart syndrome) and hemoglobin H (HbH) disease are the two most significant forms of α-thalassemia seen in clinics. Those with hemoglobin H disease require occasional or even periodic transfusions due to chronic hemolytic anemia of variable severity ranging from asymptomatic to severe forms depending on the mutations or deletions involved. While those who develop Hb Bart’s hydrops fetalis die either in *utero* or after birth due to severe intrauterine anemia.^12^

In HbH disease, the severe reduction in the synthesis of *α*-globin chains leads to a relative excess in the β-chains which accumulate in the form of b4 tetramers (HbH). Red cells containing significant amount of HbH tetramers are sensitive to oxidative stress and much more susceptible to hemolysis. Aged erythrocytes contain more precipitated HbH than younger erythrocytes and prematurely removed from the circulation. Thus, HbH disease is considered as a hemolytic disorder.^13^-^15^

Diagnosis of HbH disease depends on laboratory tests includes CBC, hemoglobin and molecular testing. Patients have characteristic parameters such as MCV, MCH in low range while very wide RDW is present. Golf balls or inclusion bodies can be observed in most of the RBCs after treating with staining dyes such as methylene blue or brilliant cresyl blue.^16^

The common *α* thalassemia deletion –α^3.7^ and the–α^4.2^ are routinely checked in most of the laboratory but do not have any data about other deletions in Pakistani population. This study aimed to find out α thalassemia deletions −−^SEA^, −−^FIL^, −−^THAI,^ —^MED^ and ––*α*^(20.5)^ in our population. Finding prevalence of these deletions help in designing different treatment options and also play important role in prevention and control of diseases. This is first report from Pakistan for the determination of prevalence of various *α*-thalassemia mutation/deletions with β-thalassemia major patients by using extended panel.

## METHODS

### Ethical approval

Research protocol was approved by the Institutional Review Board (ERC/IRB) with assigned ID code is: THL026/ NIBD/RD-16 and conformed to the tenets of the Declaration of Helsinki. This study was conducted in accordance with Good Clinical Practice (ICH GCP). Written informed consent was obtained from thalassemia patients and their parents.

### Patient recruitment and data collection

A total of 156 blood samples were collected from thalassemia patients between November 2014 and March 2016 from the National Institute of Blood Diseases and Bone Marrow (NIBD). Out of 156 patients, 97 were males and 59 were females with the ages ranging from four days to 30 years respectively. Study cohort included 121 samples of β-thal major, nine samples of β-thal minor and 26 without any evidence of β-thal mutations. Laboratory tests were performed for diagnosis of β thalassemia include CBC, Hb electrophoresis and mutational screening. Patients without any of these β-globin gene mutations were transfusion dependent and found to have high fetal hemoglobin (HbF).

### Mutation analysis

Blood samples were collected in EDTA vacutainers tubes. Genomic DNA was extracted using the Qiagen extraction kit. The multiplexed assays for detecting β-thalassemia mutation were optimized according to Suhaib et al.^17^ The primers for determination of different subsets of α-thalassemia deletion have been extensively described in the literature.^18^ The Gap polymerase chain reaction (Gap PCR) was performed in a total reaction volume of 10 μl, containing 1×PCR buffer, 25 mM Magnesium Chloride, 0.2 mM dNTPs (Fermentas), 0.25 units of Taq DNA polymerase (Merk) and 20 ng of the genomic DNA. The reaction mixture was amplified for 30 cycles with denaturation at 94 °C for one minute, annealing at 56 °C for one minute and extension at 72 °C for two minute & 10 minutes using a Veriti (Applied Biosystems, USA) for –α^3.7^ and –α^4.2^ and annealing temperature was 60°C for extended panel deletions. The PCR products were analyzed by electrophoresis on a 2% agarose gel at 120 Volts for 60 minutes (Fig.1A & B).

**Fig.1A:**
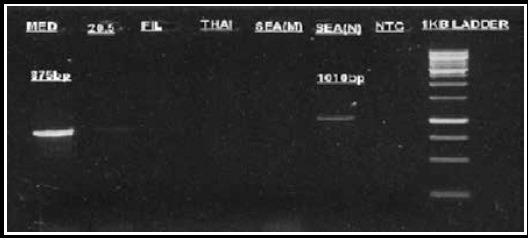
Detection of α-thalassemia −−^SEA^, −−^FIL^, −−^MED^, −−^20.5^, −−^THAI^ deletions by using 2% agarose gel stained with ethidium bromide and photographed with automated gel documentation system. Lane 1: –α^MED^ deletion (875 bp), Lane 6: Positive control for −α^SEA^ (1010 bp), Lane 8: Negative control, Lane 9: 1Kb molecular weight marker.

**Fig.1B:**
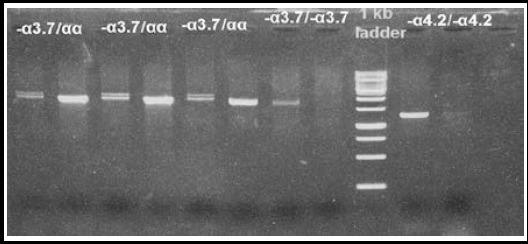
Detection of α-thalassemia α^3.7^, α^4.2^ deletions by using 2% agarose gel; Lane 1-6: Single α^3.7^deletion (2100+1900, 1900), Lane 7-8: double -α^3.7^ deletion (1900bp), Lane 9: 1Kb marker, Lane 10-11: double -α^4.2^ deletion (2100bp).

## RESULTS

**Fig.2:**
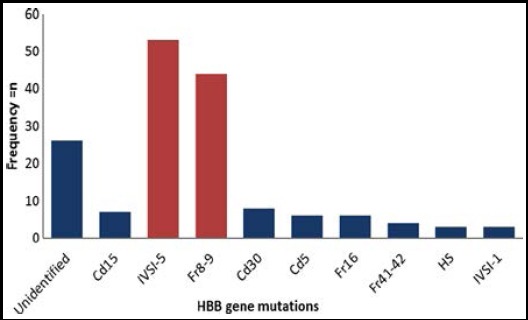
Spectrum of HBB gene mutations in β-thalassemia patients. This graph shows spectrum of all mutations found in beta thalassemia patients. IVSI-5 and Fr8-9 are the most common mutations found in Pakistani cohort.

About 121 patients were classified as β-thal major and 9 patients were β-thal minor. The mutation screening for β-thalassemia panel result showed IVSI-5 was present in 53 patients, Fr 8-9 in 44 patients, Fr 41-42 in four patients, Cd5 in 6 patients, Cd15 in 7 patients, Cd30 in 8 patients, Fr-16 in 6 patients and IVSI-1 in three patients. There was no mutation identified in 26 patients. These results indicate that IVSI-5 and Fr 8-9 are the common mutations found in β-thalassemia patients as shown in Fig.2 and Table-I. This β-thal mutations panel specifically designs for common mutation reported in Pakistani population.

**Table-I:** β Globin genotype and β thalassemia frequencies in Pakistani β-thalassemia patients.

*Genotype*	*Frequency, n*	*Percentages (%)*
Cd15	7	4.4
IVSI-5	53	33.1
Fr8-9	44	27.5
Cd30	8	5.0
Cd5	6	3.8
Fr16	6	3.8
Fr41-42	4	2.5
HS	3	1.9
IVSI-1	3	1.9
Undetermined	26	16.3

Total	160	100.0

In this study, we observed co-inheritance of α-thalassemia with homozygous β-thal in 30% cases of our cohort (37 out of 121). 27 subjects (22%) had single –α^3.7^ deletion, 4 subjects (3%) had double –α^3.7^deletion, 4 subjects (3%) had –ααα^anti3^deletion while 2 subject (2%) had showed –α^4.2^ deletion. The most common was –α^3.7^ deletion (36/38) as single/double deletions or in combination with -ααα^anti3.7^ presented in Table-II.

**Table-II:** α Globin genotype and α thalassemia frequency of α thalassemia deletion frequencies in β thalassemia major patient.

*Genotype*	*Frequency, n*	*Percentages (%)*
–α^3.7^single gene	27	22.3
–α^3.7^double gene	4	3.3
–α^4.2^single gene	2	1.7
α α α anti triplicate	4	3.3
Undetermined	84	67.8
	121	100.0

Interestingly, some patients had no alpha and beta deletion/mutation but presented with severe anemia. An extended alpha deletion panel was used to further screen these cases for any possible associated deletions. A band of Mediterranean (–α^MED^) deletion showing of approximately 875 bp was observed in two patients (Fig.1A). This will be the first study to report this mutation from indigenous Pakistani patients.

## DISCUSSION

This study was performed to determine the prevalence of α-thalassemia gene deletion in β-thalassemia patients. Co-inheritance of α-thal mutation plays significant role in modifying the clinical course of homozygous β-thalassemia. Wainscoat et al. showed that coinheritance of alpha thalassemia with homozygous beta thalassemia resulted in melioration of the beta thalassemia.^19^ Several studies have reported that clinical picture of the disease changed due to the interaction of α-thal deletions with homozygous β-thal mutations.^20^, ^21^

Co-inheritance of α-thalassemia (–α^3.7,^ –α^4.2^) with homozygous β-thal was identified in 30% cases of our cohort. The most common type of deletion was –α^3.7^ and less common type were –α^4.2^ and –ααα^anti3^ which is in line with similar observation was also reported by another group from our center (NIBD). They revealed that the most prevalent *α*-thal genotype was –α^3.7^ deletion and less common type was –α^4.2^ deletions. The group of beta thalassemia patients with co-inheritance is likely to respond favorably with drugs like hydroxyurea (data not shown). Xu et al. also reported higher prevalence rate of α thalassemia carrier (8.53%), β thalassemia (2.54%) and both α and β thalassemia (0.26%) in the Guangdong Province of China. They identified two novel mutations that leads to α thalassemia, one deletion resulting in β thalassemia, and a rare deletion (_ _THAI allele) previously unreported in mainland China.^22^

Wee et al.(2008) found that incidence of α-thal in the β-thal carriers in Malaysian population was 12.7% (41/322), with the frequency of carrier was 7.8% of the SEA deletion, 3.7% of the –α^3.7^, 0.9% of Hb Constant Spring and 0.3% of the –α^4.2^.^23^ Fallah et al. also reported that –α^3.7^ was the most frequent α-globin mutation, co-inheritance of –ααα^anti3^ with β-thal frequently occurred in general population of the Iran and has adverse clinical manifestation in β-thalassemia.^24^

**Fig.3:**
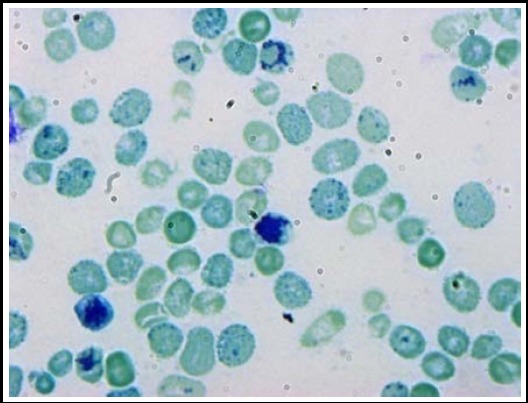
HbH inclusions within typical ‘golf ball’ cells observed in more than 30% RBCs by staining with 1% brilliant cresyl blue confirming the presence of HbH.

In our study, we found cases of hypo chromic microcytic anemia. Fetal Hb band on conventional electrophoresis was not observed in these cases. DNA mutations for β -thal mutation and α-thal deletions (–α^3.7^, –α^4.2^& –ααα^anti3.7^) were also negative. When HBH preparation was done for such patients, numerous inclusion bodies were observed on slide (Fig.3). Such cases are diagnostic dilemma and have been reported to harbor other α-thal mutation. This observation suggested the introduction of extended α-thal genetic mutation panel to find out other α-thal deletions in undiagnosed cases. We detected Mediterranean (–α^MED^) deletion at specifically 875 bp. This result gives us clue to solve these cases and revealed the correct diagnosis of HbH diseases.

–α^MED^ deletion is the most commonly found deletion in the Mediterranean region. Among 78 Cypriot patients with HbH disease, 79% had the (–α^MED^) deletion and 17% had the −α^20.5^deletion.^25^ Surprisingly, we found (–α^MED^) deletion in few cases (1.7%) during α thal extended panel screening. Some other deletions might also be found associated with HbH diseases in our population. This is a unique finding since this (–α^MED^) deletion has never been reported from Pakistan. After this finding we propose that extended panel should be used for all thalassemia patients as routine diagnostic test in Pakistan.

This study has revealed the presence of HbH disease in local population. Basic screening test like HbH inclusion body preparation should always be considered in patients with persistent anemia with hypochromic microcytic picture. Early detection and confirmation by mutation will be helpful in the treatment of disease and as well possible prevention of patients to undergo complications, such as worsening of anemia that may require red cell transfusion, cholelithiasis and iron overload. Molecular screening of HbH disease is also helpful in prenatal diagnosis.^26^

## CONCLUSION

In conclusion, α thalassemia deletions (–α^3.7,^ –α^4.2^) are the common co-inherited deletions found in beta thalassemia patients. Children presenting anemia with suspicion of haemoglobinopathy should be screened for extended alpha thalassemia genetic mutations panel. Further studies with larger cohort are required to find out the prevalence of extended genotype of α-thalassemia in Pakistani population and also clinical behavior of these patients. These observations might leads to some exciting finding for future studies.
